# Machine learning-based prediction of carbapenem-resistant *Klebsiella pneumoniae* infection risk and prognosis

**DOI:** 10.3389/fphar.2026.1780002

**Published:** 2026-03-23

**Authors:** Jiaran Sun, Linlin Yan, Ruifeng Yang

**Affiliations:** Department of Clinical Laboratory, Peking University Shougang Hospital, Beijing, China

**Keywords:** carbapenem resistance, *Klebsiella pneumoniae*, machine learning, prediction model, risk factors

## Abstract

**Objectives:**

This study aims to systematically analyze the risk factors for to carbapenem-resistant *Klebsiella pneumoniae* (CRKP) infection and its impact on patient outcomes using the XGBoost machine learning algorithm, and to explore its resistance characteristics, thereby providing a basis for early clinical identification of high-risk patients and optimizing treatment strategies.

**Methods:**

A single-center retrospective cohort study was conducted, including 491 patients with *Klebsiella pneumoniae* infections between January and December 2023. Based on drug susceptibility results, patients were divided into a resistant group (n = 187) and a susceptible group (n = 304). Univariate and multivariate logistic regression analyses were used to identify independent risk factors, and both logistic regression and XGBoost machine learning prediction models were constructed to evaluate their predictive performance and clinical applicability.

**Results:**

Patients in the resistant group were older, had worse inflammatory and coagulation indicators, and exhibited significantly higher mortality rates from 30 to 180 days compared to the susceptible group (*P* < 0.01). Multivariate logistic regression identified age, hemoglobin, lymphocyte percentage, prothrombin time, and creatinine as independent risk factors for CRKP infection, with an area under the curve (AUC) of 0.878. The XGBoost model further identified age, albumin, D-dimer, creatinine, and uric acid as key variables, with an AUC of 0.978, demonstrating superior predictive performance. Additionally, multivariate logistic regression determined age and mean platelet volume as independent risk factors for 90-day mortality in CRKP-infected patients. Based on the XGBoost algorithm, a prognostic prediction model was constructed, incorporating five key variables: age, D-dimer, creatine kinase, procalcitonin, and mean platelet volume. Drug susceptibility analysis revealed high resistance rates of CRKP to most antibiotics, with partial sensitivity only to amikacin, chloramphenicol, and cotrimoxazole.

**Conclusion:**

The XGBoost machine learning-based prediction model effectively identifies the risk of CRKP infection and poor prognosis using five key variables (age, albumin, D-dimer, creatinine, and uric acid) for infection risk, demonstrating high clinical utility and providing data support for early intervention and individualized treatment.

## 1 Introduction

*Klebsiella pneumoniae* (KPN) is a significant opportunistic pathogen responsible for both hospital-acquired and community-acquired infections. It can cause a range of serious diseases, including pneumonia, bloodstream infections, and urinary tract infections, posing a substantial threat particularly to the elderly, immunocompromised individuals, and critically ill patients (Teng et al., 2024). In recent years, with the extensive use of carbapenem antibiotics, the global prevalence of carbapenem-resistant *Klebsiella pneumoniae* (CRKP) has risen sharply, making it one of the most critical challenges in the field of global public health (Lei et al., 2024).

Compared to infections caused by carbapenem-sensitive *Klebsiella pneumoniae* (CSKP), CRKP infections are associated with limited treatment options and extensive multidrug resistance. This often leads to clinical treatment failure, prolonged hospitalization, significantly increased healthcare costs, and ultimately a substantial rise in patient mortality (Budia-Silva et al., 2024). Currently, the early identification of CRKP infections primarily relies on microbiological culture and antimicrobial susceptibility testing, the results of which are often delayed, potentially missing the optimal window for intervention (Lee et al., 2023). Therefore, it is crucial to rapidly and accurately identify patients at high risk of CRKP infection in the early stages and predict adverse outcomes using available clinical and laboratory indicators. This approach is essential for guiding early implementation of effective infection control measures, optimizing initial empirical antibiotic therapy, and ultimately improving patient prognosis.

Recent studies have increasingly applied machine learning and nomogram techniques to predict infections and outcomes in surgical or intensive care settings, demonstrating the growing potential of these approaches for early risk stratification (Xu et al., 2025; Lahariya et al., 2026; Fan et al., 2022; Lahariya et al., 2025). Although previous studies have explored risk factors for CRKP infections, most have focused on single indicators or traditional logistic regression models (Yang et al., 2024; Zhou et al., 2024). These studies have not sufficiently integrated multi-system and multi-dimensional clinical information and lack the application of more advanced machine learning algorithms to uncover complex patterns within the data and construct high-precision predictive models. Furthermore, a comprehensive assessment of the antimicrobial susceptibility profiles of CRKP-infected patients and their association with host clinical characteristics is crucial for guiding the rational use of antibiotics in the local setting.

Building on this background, the present study aims to systematically analyze the differences in clinical characteristics, laboratory indicators, and outcomes between patients infected with CRKP and those infected with CSKP. By integrating traditional statistical methods with the machine learning algorithm eXtreme Gradient Boosting (XGBoost), this research will develop and validate predictive models for CRKP infection and prognosis. Additionally, the study will provide a detailed description of the resistance profiles of CRKP strains. The findings are expected to offer robust data support and a basis for clinical decision-making, facilitating early identification of high-risk patients, the formulation of precise individualized treatment strategies, and the optimization of hospital antimicrobial stewardship.

## 2 Materials and methods

### 2.1 Study design and participants

This study is a single-center, retrospective cohort study. We consecutively enrolled a total of 491 inpatients at the hospital diagnosed with KPN infection by microbiological testing at our hospital between January 2023 and December 2023. Outpatients and emergency department patients without subsequent admission were not included. The inclusion criteria were: age ≥18 years; positive laboratory test specimens for KPN, meeting the criteria for healthcare-associated infections set by the Centers for Disease Control and Prevention (Horan et al., 2008). Exclusion criteria were: lack of key data; patients with co-infections involving other bacteria; and laboratory-identified non-pathogenic (colonizing) strains. For patients readmitted, only the first infection episode was retained. This study was reviewed and approved by the Ethics Committee of Peking University Shougang Hospital (No. IRBK-2024-026-01).

Based on antimicrobial susceptibility test results, all patients were divided into two groups: the CRKP group (n = 187) and CSKP group (n = 304).

### 2.2 Data collection

By reviewing the Hospital Information System (HIS) and Laboratory Information System (LIS), two researchers independently collected patient demographics, laboratory test indicators, antimicrobial susceptibility test results, clinical outcomes, and other relevant information. A comprehensive list of all parameters collected during the study is provided in Supplementary Table S1.

### 2.3 Specimen requirements and antimicrobial susceptibility testing methods

For complete blood count, venous blood samples collected within 24 h of admission were required as baseline data. For inpatients, specimens were collected daily in the morning while fasting or as clinically indicated. For biochemical indicators such as liver function, venous blood was uniformly collected after an 8- to 12-h overnight fast to eliminate interference from diet and physiological rhythms. All testing was performed using standardized automated analyzers in the clinical laboratory of our hospital.

Antimicrobial susceptibility testing was performed and interpreted according to the latest guidelines published by the Clinical and Laboratory Standards Institute. Minimum inhibitory concentrations (MICs) were determined using either the agar dilution or broth microdilution method, supplemented by the disk diffusion method for routine rapid testing. All results were interpreted as susceptible, intermediate, or resistant according to CLSI standards (Humphries et al., 2021). Standard quality control strains were used concurrently to ensure the accuracy and reliability of the experimental results.

### 2.4 Statistical analysis

All statistical analyses and model developments were performed using R software (version 4.2.2) and SPSS (version 26.0). A comprehensive list of all R packages employed, along with their versions and selection rationales, is provided in Supplementary Table S2. Normally distributed continuous data are presented as mean ± standard deviation (x ± s), and comparisons between groups were made using the independent samples t-test. Non-normally distributed continuous data are presented as median (interquartile range) [M (IQR)], and the Mann-Whitney U test was used for group comparisons. Categorical data are presented as number (percentage) [n (%)], and comparisons were made using the chi-square test or Fisher’s exact test. For the Antimicrobial Susceptibility Analysis the intermediate and resistant categories were combined into a “Non-susceptible” group for comparison. Chi-square tests were not performed for antimicrobial agents with zero observed cases. A *P*-value <0.05 was considered statistically significant.

To identify independent risk factors for CRKP infection, univariable logistic regression analysis was first performed on all variables, calculating odds ratios (ORs) and their 95% confidence intervals (CIs). Based on the independent risk factors screened from the univariable analysis, a multivariable logistic regression prediction model was constructed. The model’s discriminatory ability was evaluated using the receiver operating characteristic (ROC) curve, and the area under the curve (AUC) was calculated. A calibration curve was plotted to assess model calibration. The model’s clinical net benefit was evaluated using decision curve analysis (DCA).

The XGBoost algorithm was employed for model development. Hyperparameters were tuned using a grid search strategy combined with 5-fold cross-validation to ensure robust model generalizability. In this approach, the dataset was randomly partitioned into five subsets; the model was iteratively trained on four folds and validated on the remaining fold. The optimal hyperparameter combination was selected based on the lowest average cross-validated log loss. The final model was then retrained on the full training dataset using the selected parameters, with early stopping implemented to prevent overfitting. All laboratory indicators were ranked by feature importance using SHapley Additive exPlanations (SHAP) values, with the top five important variables selected. SHAP values were employed to assess the contribution of features to the model output, enabling both the evaluation of global feature importance (based on the mean absolute value across all samples) and the interpretation of individual predictions at the single-sample level. Subsequently, an XGBoost prediction model was constructed based on these selected variables and its performance was evaluated. The same statistical analysis workflow was applied for predicting the 90-day mortality risk in CRKP patients.

## 3 Results

### 3.1 Clinical and demographic characteristics of patients

Between January 2023 and December 2023, a total of 491 patients with KPN infection were finally included in this study. Among them, 187 cases (38.1%) were CRKP infections, and 304 cases (61.9%) were CSKP infections. A comparison of complete blood count, liver function, renal function, cardiac markers, electrolytes and metabolic indicators, inflammatory markers, and coagulation function between the two groups revealed statistically significant differences in all the aforementioned parameters (*P <* 0.05, Table 1).

**TABLE 1:** Demographic and laboratory parameters of KPN patients.

Index	CSKP (n = 304)	CRKP (n = 187)	*P*
Age, year	55 (46–67)	78 (65–87)	<0.001
Male, n (%)	116 (38.2)	71 (38.0)	0.961
Blood routine			
RBC (×10^12^/L)	3.80 (3.14–4.32)	3.18 (2.63–3.72)	<0.001
WBC (×10^9^/L)	7.60 (5.50–10.38)	9.40 (6.70–14.10)	<0.001
PLT (×10^9^/L)	203.00 (146.00–274.50)	197.00 (120.00–271.00)	0.179
MON (%)	6.30 (4.50–7.90)	5.70 (3.60–7.50)	0.078
LYM (%)	16.65 (8.63–26.35)	10.90 (6.20–17.90)	<0.001
EOS (%)	1.00 (0.20–2.58)	0.90 (0.00–2.80)	0.475
BAS (%)	0.30 (0.20–0.50)	0.30 (0.10–0.40)	0.024
NEU (%)	73.00 (60.53–83.70)	79.10 (69.80–88.40)	<0.001
HGB (g/L)	115.00 (95.00–131.00)	94.00 (80.00–113.00)	<0.001
HCT (%)	33.90 (28.93–38.88)	29.00 (24.00–34.00)	<0.001
MCH (pg)	30.50 (29.20–31.48)	30.60 (29.20–31.90)	0.157
MCHC (g/L)	338.00 (328.00–345.00)	335.00 (326.00–342.00)	0.009
MCV (fL)	89.90 (86.60–93.08)	91.90 (87.40–95.90)	0.005
MPV (fL)	10.20 (9.70–11.00)	10.80 (9.90–11.50)	<0.001
PDW (fL)	11.30 (10.20–12.90)	12.40 (10.50–14.10)	0.001
RDW (%)	13.50 (12.70–14.90)	14.70 (13.30–16.50)	<0.001
Liver function			
ALT (U/L)	18.00 (11.00–29.00)	18.00 (11.00–35.00)	0.497
AST (U/L)	21.50 (17.00–33.75)	28.00 (20.00–47.00)	<0.001
ALB (g/L)	36.80 (32.63–40.68)	32.70 (29.90–34.90)	<0.001
TP (g/L)	63.30 (56.28–68.43)	59.30 (54.10–64.20)	<0.001
TBIL (μmol/L)	11.00 (7.35–15.70)	9.20 (6.30–14.80)	0.057
Renal function			
UA (μmol/L)	286.00 (198.00–361.50)	236.00 (147.00–373.00)	0.015
UREA (mmol/L)	5.86 (4.08–8.50)	9.14 (5.13–15.07)	<0.001
CREA (μmol/L)	66.40 (53.43–80.97)	73.60 (50.50–143.20)	0.005
Cardiac biomarkers			
LDH (U/L)	179.00 (149.00–231.25)	218.00 (159.00–336.00)	<0.001
HBDH (U/L)	125.00 (105.25–164.75)	162.00 (117.00–231.00)	<0.001
CK(U/L)	53.00 (26.25–100.00)	40.00 (22.00–95.00)	0.172
CK-MB(U/L)	12.00 (9.00–17.00)	12.00 (8.00–20.00)	0.832
Electrolytes and metabolic panel			
GLU (mmol/L)	6.80 (5.47–9.42)	7.87 (5.91–11.23)	0.002
K (mmol/L)	3.85 (3.52–4.23)	3.93 (3.60–4.31)	0.059
Na (mmol/L)	137.00 (134.25–140.00)	136.00 (132.00–140.00)	0.267
CO2CP(mmol/L)	22.50 (20.40–24.60)	23.10 (20.20–26.00)	0.170
Inflammatory markers			
PCT (ng/mL)	0.26 (0.18–0.36)	0.38 (0.22–1.15)	<0.001
IL-6 (pg/mL)	21.90 (6.70–71.45)	38.60 (15.00–89.60)	<0.001
CRP (mg/L)	21.65 (4.44–73.27)	42.24 (18.13–102.87)	<0.001
Coagulation panel			
PT (s)	12.50 (11.80–13.80)	13.40 (12.40–14.50)	<0.001
PT-INR	1.06 (1.00–1.19)	1.15 (1.06–1.24)	<0.001
PTA (%)	84.90 (73.05–93.15)	76.50 (65.60–86.80)	<0.001
TT (s)	16.80 (16.10–17.50)	16.80 (16.10–18.00)	0.499
APTT (s)	28.30 (24.63–34.30)	32.10 (27.10–39.40)	<0.001
AT3 (%)	79.80 (69.28–91.75)	74.50 (63.30–84.30)	<0.001
D-D (mg/L)	1.70 (0.70–3.88)	3.20 (1.70–6.10)	<0.001
FBG (g/L)	3.60 (2.90–4.70)	3.90 (2.90–5.10)	0.222
FDP (mg/L)	3.30 (1.60–8.10)	6.00 (3.60–12.90)	<0.001

Abbreviations: ALB, albumin; ALT, alanine aminotransferase; APTT, activated partial thromboplastin time; AST, aspartate aminotransferase; AT3, antithrombin III; BAS%, basophil percentage; CK, creatine kinase; CK-MB, creatine kinase isoenzyme-MB; CO2CP, carbon dioxide combining power; CREA, creatinine; CRP, C-reactive protein; CSKP, carbapenem-sensitive *Klebsiella pneumoniae*; CRKP, carbapenem-resistant *Klebsiella pneumoniae*; D-D, D-dimer; EOS%, eosinophil percentage; FBG, fibrinogen; FDP, fibrinogen degradation products; GLU, glucose; HBDH, hydroxybutyrate dehydrogenase; HCT, hematocrit; HGB, hemoglobin; IL-6, interleukin-6; K, potassium; LDH, lactate dehydrogenase; LYM%, lymphocyte percentage; MCH, mean corpuscular hemoglobin; MCHC, mean corpuscular hemoglobin concentration; MCV, mean corpuscular volume; MON%, monocyte percentage; MPV, mean platelet volume; Na, sodium; NEU%, neutrophil percentage; PCT, procalcitonin; PDW, platelet distribution width; PLT, platelet; PT, prothrombin time; PTA, prothrombin time activity; PT-INR, prothrombin time-international normalized ratio; RBC, red blood cell; RDW, red cell distribution width; TBIL, total bilirubin; TP, total protein; TT, thrombin time; UA, uric acid; UREA, urea; WBC, white blood cell.

**FIGURE 1:**
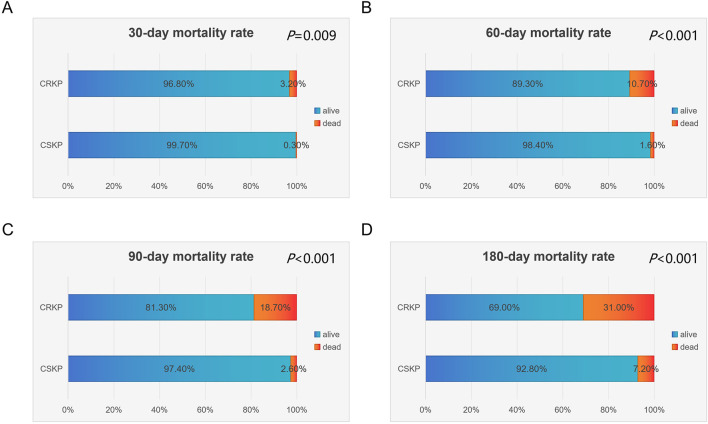
Comparison of mortality rates at various time points between CRKP and CSKP patients. **(A)** 30-day mortality, **(B)** 60-day mortality, **(C)** 90-day mortality, **(D)** 180-day mortality.

Further comparison of mortality rates at 30, 60, 90, and 180 days between the two groups is presented in Figure 1. The CRKP group exhibited significantly higher mortality rates compared to the CSKP group at all four time points: 30-day mortality (3.20% vs. 0.30%, *P* = 0.009), 60-day mortality (24.1% vs. 6.3%), 90-day mortality (18.70% vs. 2.60%, *P* < 0.001), and 180-day mortality (31.00% vs. 7.20%, *P* < 0.001). These findings demonstrate that CRKP infection is associated with substantially higher short-term and long-term mortality compared to CSKP infection, underscoring the severe clinical impact of CRKP.

### 3.2 Risk factors for CRKP occurrence

**FIGURE 2:**
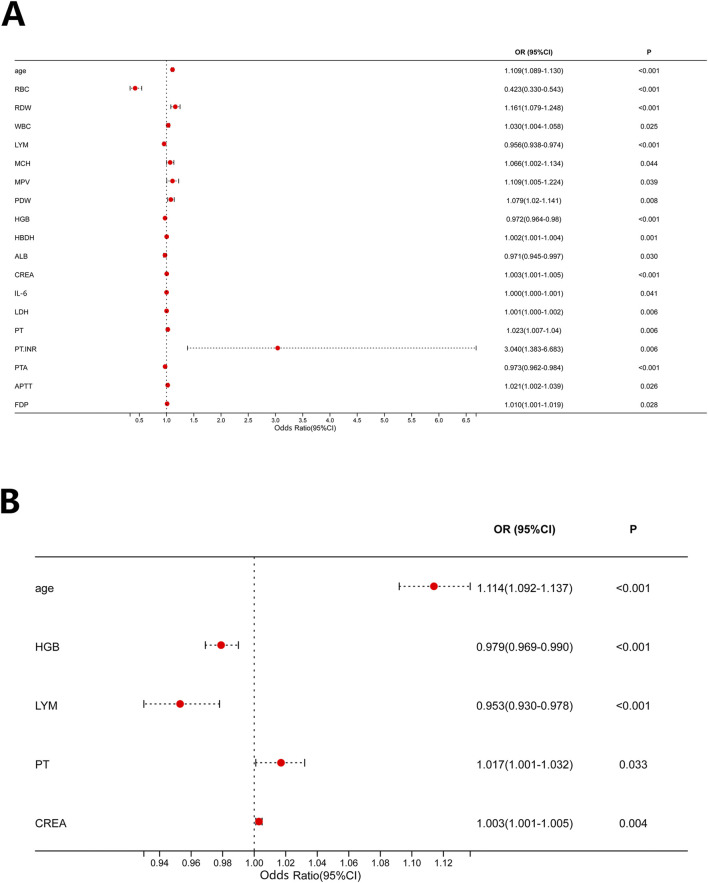
Risk factors for CRKP occurrence. **(A)** Univariable logistic regression analysis; **(B)** Multivariable logistic regression analysis. Abbreviations: ALB, albumin; APTT, activated partial thromboplastin time; CREA, creatinine; FDP, fibrinogen degradation products; HBDH, hydroxybutyrate dehydrogenase; HGB, hemoglobin; IL-6, interleukin-6; LDH, lactate dehydrogenase; LYM, lymphocyte; MCH, mean corpuscular hemoglobin; MPV, mean platelet volume; PDW, platelet distribution width; PT, prothrombin time; PTA, prothrombin time activity; PTNR, prothrombin time ratio; RBC, red blood cell; RDW, red cell distribution width; WBC, white blood cell.

Univariable logistic regression analysis was performed on all indicators, identifying a total of 19 factors significantly associated with CRKP occurrence (Figure 2A). Further multivariable logistic regression analysis of these 19 factors (Figure 2B) revealed that five variables were independently associated with CRKP infection. Among these, advanced age (OR = 1.114, 95% CI: 1.092–1.137, *P* < 0.001), prolonged PT (OR = 1.017, 95% CI: 1.001–1.032, *P* = 0.033), and elevated serum CREA (OR = 1.003, 95% CI: 1.001–1.005, *P* = 0.004) showed positive associations with CRKP infection, indicating they were risk factors. In contrast, higher hemoglobin levels (OR = 0.979, 95% CI: 0.969–0.990, *P* < 0.001) and higher lymphocyte percentage (OR = 0.953, 95% CI: 0.930–0.978, *P* < 0.001) showed negative associations with CRKP infection, indicating they were protective factors.

### 3.3 Development and evaluation of a logistic regression-based CRKP infection model

**FIGURE 3:**
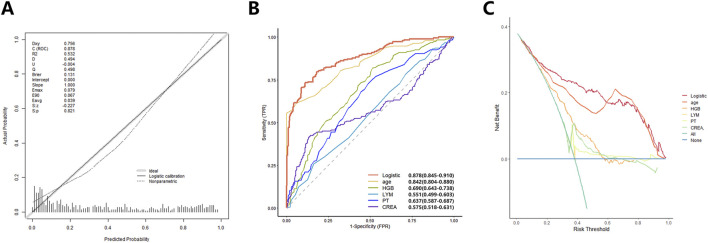
Development and evaluation of the logistic regression-based CRKP susceptibility model. **(A)** Calibration curve of the logistic model; **(B)** Comparison of ROC curves between the multi-variable logistic model and single indicators; **(C)** Comparison of Decision Curve Analysis (DCA) between the multi-variable logistic model and single indicators.

We constructed a logistic regression prediction model based on the aforementioned five independent risk factors and compared its predictive performance. The results demonstrated that the calibration curve of the logistic model showed good agreement between the predicted probabilities and the actual outcomes (Figure 3A). The logistic model achieved the largest AUC of 0.878 (95% CI: 0.845–0.910), outperforming any individual predictor (Figure 3B). DCA was performed across threshold probabilities from 0% to 100% to evaluate the clinical net benefit of the model. The analysis demonstrated that the logistic model provided positive net benefit across a wide range of clinically relevant threshold probabilities (approximately 20%–60%), suggesting potential clinical utility that warrants further validation (Figure 3C).

### 3.4 Development and evaluation of XGBoost model for CRKP infection

**FIGURE 4:**
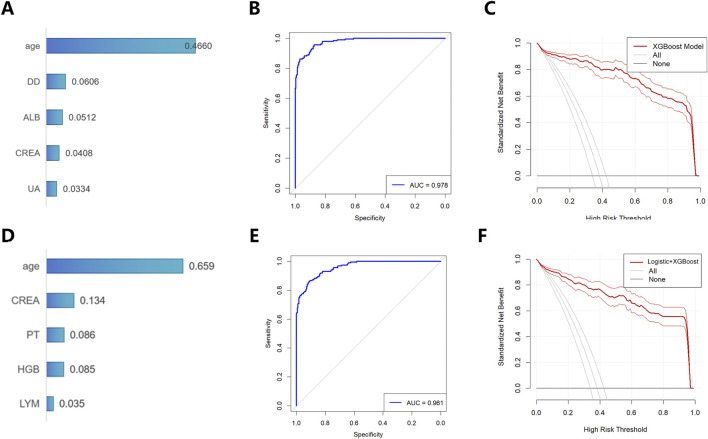
Development and evaluation of a machine learning (XGBoost) model for CRKP susceptibility. **(A)** Top five important variables related to CRKP identified by the XGBoost algorithm (ranked by SHAP values); **(B)** ROC analysis and **(C)** DCA analysis of the susceptibility model constructed using the aforementioned five variables; **(D)** SHAP values of variables from the model based on independent risk factors screened by multivariable logistic regression combined with XGBoost; **(E)** ROC analysis and **(F)** DCA analysis of this model.

We further employed the XGBoost model to analyze all laboratory indicators. Based on SHAP values for feature importance, the top five predictive factors were identified as age, ALB, D-D, CREA, and UA (Figure 4A). The XGBoost prediction model was constructed using these five variables, and its performance was evaluated using the ROC curve. The model achieved an AUC value of 0.978 (Figure 4B), indicating excellent discriminative ability. The SHAP summary plot (Figure 4A) further revealed the direction of association for each variable: higher age, elevated D-dimer, increased creatinine, and elevated uric acid were associated with higher CRKP risk, while higher albumin levels were associated with lower CRKP risk.

Furthermore, we incorporated the five indicators identified from the multivariable analysis into the XGBoost model for feature importance evaluation (Figure 4D) and established another predictive model. The ROC curve for this model corresponded to an AUC of 0.961 (Figure 4E). Comparative results demonstrated that the model directly built upon variables selected by the XGBoost model exhibited superior predictive performance. DCA showed that the XGBoost model provided greater clinical net benefit (Figures 4C,F), further substantiating its strong potential for clinical application.

### 3.5 Risk factors and prognostic model for 90-day mortality in patients with CRKP patients

**FIGURE 5:**
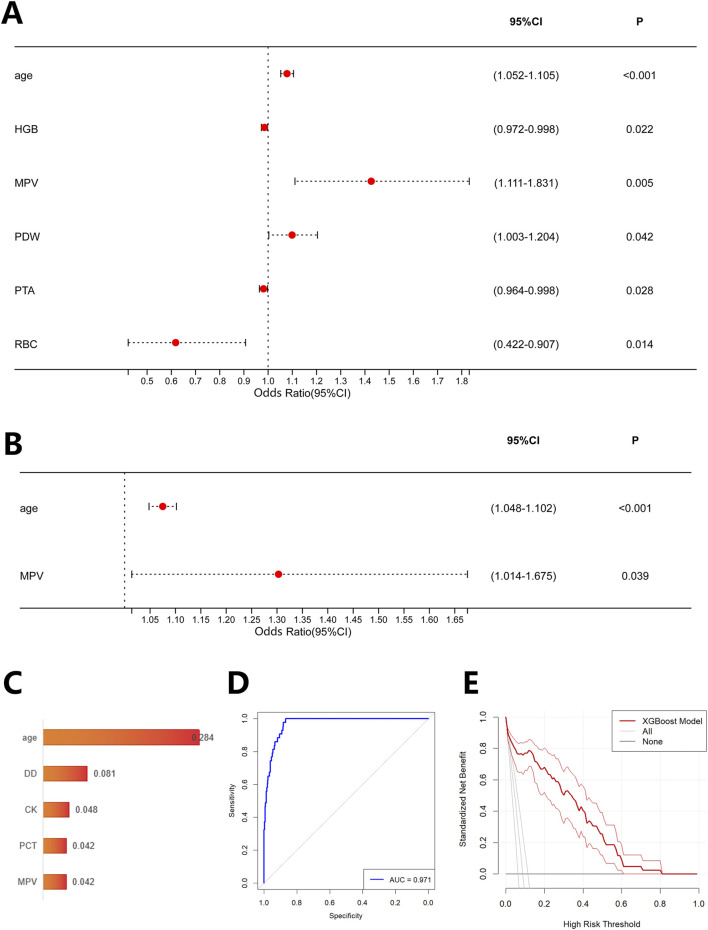
Risk factors and prognostic model for 90-day mortality in patients with KPN infection. **(A)** Univariate analysis and **(B)** multivariate analysis of risk factors for 90-day mortality in patients with KPN infection; **(C)** Top five important variables associated with 90-day mortality identified by the XGBoost algorithm (ranked by SHAP values); **(D)** ROC analysis and **(E)** DCA analysis of the prognostic model constructed using XGBoost based on the aforementioned five variables.

Based on their 90-day outcomes, CRKP patients were categorized into survival and non-survival groups. Univariate analysis (Table 2) identified several factors associated with a 90-day mortality risk in CRKP patients, including age, hemoglobin (HGB), mean platelet volume (MPV), platelet distribution width (PDW), prothrombin time activity (PTA), and red blood cell count (RBC) (Figure 5A). Multivariate analysis further indicated that advanced age (OR = 1.075, 95% CI: 1.048–1.102, P < 0.001) and elevated MPV (OR = 1.303, 95% CI: 1.014–1.675, P = 0.039) showed positive associations with 90-day mortality in CRKP patients, indicating they were risk factors (Figure 5B). The prediction model constructed using the XGBoost algorithm identified age, D-D, creatine kinase (CK), procalcitonin (PCT), and MPV as the top five important variables for predicting 90-day mortality. The SHAP analysis revealed that higher age, elevated D-dimer, increased CK, elevated PCT, and higher MPV were associated with increased mortality risk (Figure 5C). This model demonstrated excellent discriminatory ability, achieving an AUC of 0.971 (Figure 5D). DCA results indicated significant clinical net benefit and robust overall performance of the model, suggesting its high potential for clinical application (Figure 5E).

**TABLE 2:** Antimicrobial susceptibility analysis of common antibiotics in patients infected with KPN.

Antibiotic	Susceptibility result	CSKP (n = 304)	CRKP (n = 187)	*P*
Amoxicillin-clavulanic acid (AMC)	Susceptible	198 (65.13)	5 (2.67)	<0.001
	Intermediate	40 (13.16)	1 (0.53)	
	Resistant	66 (21.71)	181 (96.79)	
Amikacin (AMK)	Susceptible	300 (98.68)	111 (59.36)	<0.001
	Intermediate	0 (0.00)	2 (1.07)	
	Resistant	4 (1.32)	74 (39.57)	
Ampicillin (AMP)	Susceptible	243 (79.93)	0 (0.00)	-
	Intermediate	15 (4.93)	0 (0.00)	
	Resistant	46 (15.13)	187 (100.00)	
Aztreonam (ATM)	Susceptible	232 (76.32)	0 (0.00)	-
	Intermediate	7 (2.30)	0 (0.00)	
	Resistant	65 (21.38)	187 (100.00)	
Ceftazidime (CAZ)	Susceptible	230 (75.66)	7 (3.74)	<0.001
	Intermediate	5 (1.64)	3 (1.60)	
	Resistant	69 (22.70)	177 (94.65)	
Chloramphenicol (CMP)	Susceptible	252 (82.89)	89 (47.59)	<0.001
	Intermediate	12 (3.95)	0 (0.00)	
	Resistant	40 (13.16)	98 (52.41)	
Ciprofloxacin (CIP)	Susceptible	224 (73.68)	13 (6.95)	<0.001
	Intermediate	6 (1.97)	5 (2.67)	
	Resistant	74 (24.34)	169 (90.37)	
Cefotaxime (CTX)	Susceptible	211 (69.41)	5 (2.67)	<0.001
	Intermediate	4 (1.32)	2 (1.07)	
	Resistant	87 (28.62)	180 (96.26)	
Cefazolin (CFZ)	Susceptible	234 (76.97)	5 (2.67)	<0.001
	Intermediate	10 (3.29)	0 (0.00)	
	Resistant	60 (19.74)	182 (97.33)	
Cefepime (FEP)	Susceptible	266 (87.50)	5 (2.67)	<0.001
	Intermediate	5 (1.64)	2 (1.07)	
	Resistant	33 (10.86)	180 (96.26)	
Gentamicin (GM)	Susceptible	293 (96.38)	31 (16.58)	<0.001
	Intermediate	7 (2.30)	5 (2.67)	
	Resistant	4 (1.32)	151 (80.75)	
Imipenem (IPM)	Susceptible	213 (70.07)	0 (0.00)	-
	Intermediate	0 (0.00)	0 (0.00)	
	Resistant	91 (29.93)	187 (100.00)	
Levofloxacin (LEV)	Susceptible	298 (98.03)	14 (7.49)	<0.001
	Intermediate	3 (0.99)	23 (12.30)	
	Resistant	3 (0.99)	150 (80.21)	
Meropenem (MEM)	Susceptible	249 (81.91)	0 (0.00)	-
	Intermediate	18 (5.92)	0 (0.00)	
	Resistant	34 (11.18)	187 (100.00)	
Moxifloxacin (MXF)	Susceptible	202 (66.45)	9 (4.81)	<0.001
	Intermediate	32 (10.53)	9 (4.81)	
	Resistant	70 (23.03)	169 (90.37)	
Trimethoprim-sulfamethoxazole (SXT)	Susceptible	227 (74.67)	76 (40.64)	<0.001
	Intermediate	0 (0.00)	0 (0.00)	
	Resistant	77 (25.33)	111 (59.36)	
Tetracycline (TE)	Susceptible	222 (73.03)	17 (9.09)	<0.001
	Intermediate	6 (1.97)	24 (12.83)	
	Resistant	76 (25.00)	146 (78.07)	
Piperacillin-tazobactam (TZP)	Susceptible	281 (92.43)	10 (5.35)	<0.001
	Intermediate	8 (2.63)	1 (0.53)	
	Resistant	15 (4.93)	176 (94.12)	

### 3.6 Antimicrobial susceptibility analysis

The antimicrobial susceptibility testing results (Table 2) revealed highly significant differences in susceptibility patterns between CSKP and CRKP strains. CSKP strains maintained relatively high susceptibility to most antimicrobials tested, particularly to amikacin (98.68%), levofloxacin (98.03%), gentamicin (96.38%), and piperacillin-tazobactam (92.43%). In contrast, CRKP strains exhibited extensive multidrug resistance. All CRKP strains were completely resistant (100%) to ampicillin, aztreonam, imipenem, and meropenem. Chi-square test analysis demonstrated that the differences in susceptibility rates between the CSKP and CRKP groups were statistically significant (*P <* 0.001) for all antimicrobials tested. Although CRKP strains were resistant to most drugs, they retained relatively higher susceptibility to amikacin (59.36%), chloramphenicol (47.59%), and trimethoprim-sulfamethoxazole (40.64%), though these rates were still significantly lower than those in the CSKP group (*P <* 0.001).

## 4 Discussion

This study identified predictors of CRKP infection using multivariable logistic regression and the XGBoost model, respectively, revealing significant differences and complementarity in variable selection and predictive performance. The research encompassed two core components: the development of a risk model for CRKP infection and an investigation into post-infection prognostic modeling.

In the model, the five independent risk factors identified by logistic regression (age, HGB, LYM%, PT, CREA) delineated a comprehensive clinical profile of the host from traditional pathophysiological dimensions, including immune status, coagulation, and renal function. This model demonstrated strong performance (AUC = 0.878) and maintained good clinical interpretability. In contrast, the strength of the machine learning model lies in its ability to capture complex, non-linear relationships between variables, thereby offering more precise risk stratification. The XGBoost model (AUC = 0.978) further optimized predictive performance. Its top five important variables (age, ALB, D-D, CREA, UA) were more sensitive in capturing dynamic processes such as inflammation, thrombosis, and metabolic status, revealing a more intricate interplay of risk mechanisms. The two methods offer a three-dimensional perspective on CRKP risk from the angles of mechanism inference and data-driven analysis, respectively. Their combined use balances clinical interpretability with predictive accuracy, providing a more comprehensive basis for early intervention.

Age was identified as a core risk factor by both models, its significance extending far beyond simple chronological increase. Elderly patients face systemic physiological decline. Aging is associated with a functional decline in both innate and adaptive immune responses, a phenomenon termed immunosenescence (Zou et al., 2025). This manifests as reduced T-lymphocyte diversity and dysfunction, weakening the ability to respond to novel pathogens such as drug-resistant bacteria, alongside diminished neutrophil chemotaxis and phagocytic capacity (Hariyanto et al., 2021). Consequently, the aged host is less effective at recognizing and clearing CRKP, leading to a higher likelihood of severe infection progression. Furthermore, elderly individuals often present with multiple coexisting chronic conditions, such as diabetes, cardiovascular disease, chronic obstructive pulmonary disease (Salaffi et al., 2021). These underlying diseases not only impair organ function, but their treatment processes also significantly increase the risk of exposure to and colonization by drug-resistant bacteria, such as through frequent hospitalizations, invasive procedures, and prolonged antibiotic exposure (Capsoni et al., 2025). Aging leads to a sharp decline in functional reserve across organ systems. Under the stress of infection, elderly patients are more prone to decompensation, rapidly progressing to multiple organ failure with a consequently poor prognosis.

CREA, elevated levels of which directly indicate renal impairment, is another key biomarker highlighted by both models. Studies have shown that acute renal failure is a risk factor for the development of CRKP infection (Akgul et al., 2016). Severe infection itself can induce acute kidney injury (AKI) through sepsis-related hypoperfusion and cytokine storm (Dou et al., 2021). This indicates that there is a bidirectional pathophysiological link between acute renal insufficiency and CRKP infection. Consequently, CREA, representing renal function, has a logical basis as a potential early warning signal for CRKP infection; its elevation may indicate a high-risk patient status or infection with a more virulent pathogen. Changes in CREA levels may be both a consequence of CRKP infection and a predictor of its risk, suggesting its potential utility as a clinical indicator. However, due to its limited specificity, CREA should be incorporated into a multifactor comprehensive assessment system rather than being used as a standalone diagnostic predictor. In the XGBoost model, the inclusion of UA may be associated with the progression of pre-existing renal disease and increased mortality resulting from the development of hyperuricemia (Suliman et al., 2006; Syrjänen et al., 2000). The XGBoost model is capable of capturing nonlinear relationships and interaction effects among variables, which is particularly relevant given that the association between UA and CRKP risk may exhibit nonlinear patterns (e.g., a U-shaped relationship), a complexity that traditional linear models struggle to accommodate. Consequently, XGBoost offers enhanced sensitivity in identifying UA as a potential predictor, underscoring its early-warning value in the context of metabolic disturbances or renal impairment.

LYM% and ALB, selected by the logistic regression and XGBoost models respectively, jointly delineate the host’s intrinsic resistance capacity, offering two complementary perspectives on immuno-nutritional status. LYM% reflects cellular immune function. Lymphocytes are central to combating intracellular pathogens and orchestrating specific immune responses (Lv et al., 2024). A decreased LYM% may indicate suppressed or weakened cellular immunity, leading to reduced immune clearance capacity and potentially impairing the host’s ability to eliminate CRKP. This is commonly observed in patients with sepsis, those undergoing chemotherapy, or individuals with inherent immunodeficiencies, and serves as a strong predictor of poor prognosis (Yu et al., 2022; Chen et al., 2022; Pei et al., 2022). ALB reflects host nutritional and inflammatory status (Ji et al., 2023). Hypoalbuminemia is far more than a simple nutritional issue; it is a potent negative acute-phase reactant (Wu et al., 2023). Research indicates that during severe infection and inflammation, cytokines (such as IL-6) suppress hepatic albumin synthesis, while increased vascular permeability contributes to its extravasation (Xu et al., 2023). Therefore, low ALB is a composite marker of disease severity, persistent inflammation, and malnutrition. Malnutrition further impairs the function and proliferative capacity of immune cells, creating a vicious cycle with lymphopenia that collectively leads to immune paralysis.

PT and D-D, both coagulation-related indicators, exemplify the models’ progression from routine screening to sensitive biomarkers. PT reflects the functional state of the extrinsic coagulation pathway. Prolonged PT indicates consumptive reduction or insufficient synthesis of coagulation factors (e.g., II, V, VII, X) (Adcock et al., 2023). In severe infections, pathogens and the ensuing inflammatory response can extensively activate the coagulation system, forming microthrombi and consuming substantial clotting factors, resulting in PT prolongation. It serves as a marker of severe infection and potential progression toward disseminated intravascular coagulation (DIC), albeit with relatively lower sensitivity. D-D reflects the dynamic process of fibrinolysis activation and thrombosis. D-D is a specific degradation product of fibrin. A significant elevation in its levels indicates widespread fibrin formation and degradation within the body, signifying active thrombotic processes (Tayal et al., 2024). In infections with gram-negative bacteria such as CRKP, endotoxemia and inflammation readily cause endothelial cell damage, triggering microvascular thrombosis (Tang et al., 2021). Consequently, D-D may serve as a more sensitive indicator of infection-related coagulation dysregulation compared to traditional coagulation tests such as PT. This is supported by recent studies demonstrating that D-D elevations often precede abnormalities in conventional coagulation parameters and exhibit stronger associations with disease severity. For instance, a study of children with severe pneumonia found that while PT, APTT, and FIB were elevated in severe cases, D-D and FDP levels were negatively correlated with pediatric critical illness scores and showed greater discriminatory power for prognosis (Song et al., 2024). Similarly, research on sepsis stratification has shown that D-D, when combined with inflammatory markers, enhances diagnostic accuracy for identifying progression to septic shock, with D-D demonstrating a sensitivity of 76.6% compared to 53.2% for PT (Ding et al., 2026). Experimental models of prothrombotic imbalance have also confirmed that markers such as D-dimer are substantially more sensitive to early coagulation disturbances than traditional APTT/PT/fibrinogen measures (Brooks et al., 2025). These findings collectively indicate that D-D reflects the dynamic process of fibrinolysis activation and thrombosis, offering a more sensitive and earlier window into infection-related endothelial injury and coagulation dysregulation. This likely explains its superior predictive value in the XGBoost model compared to PT.

Our findings complement recent CRKP prediction studies from two perspectives. Yao et al. (2024) identified clinical exposures such as hospitalization, antibiotics, and procedures as key predictors, whereas our XGBoost model highlighted host physiological markers together capturing both external risk and internal susceptibility. Alparslan et al. (2025) found prolonged ICU stay to be the dominant predictor in an ICU cohort. In their high-exposure setting, variability in exposure duration drove risk. In our more heterogeneous inpatient population, where exposure intensity varies widely, host physiological status emerged as the superior discriminator. Together, these findings suggest that optimal risk models should be tailored to specific clinical scenarios: exposure-focused for ICUs; and physiology-focused for general wards.

This study’s systematic analysis of 491 patients with KPN infection reaffirmed that CRKP is a critical risk factor for worse patient outcomes: mortality rates at all time points from 30 to 180 days were significantly higher in the CRKP group compared to the CSKP group. Building on this, we further identified risk factors associated with mortality in CRKP-infected patients through prognostic model analysis. Multivariable analysis confirmed advanced age and elevated MPV as independent risk factors. This aligns perfectly with the central role of advanced age in the portrait of CRKP-susceptible individuals, suggesting immunosenescence as the cornerstone determining infection outcomes. The inclusion of MPV provides a novel mechanistic perspective. MPV is an indirect indicator of platelet size and activity, and elevated MPV has been associated with pro-inflammatory and prothrombotic states in various clinical conditions (Emre et al., 2020; Jiang et al., 2020). Although MPV does not directly measure inflammation or thrombosis, its elevation may reflect underlying platelet activation, which can contribute to the pathogenesis of sepsis-related coagulation abnormalities. During sepsis, abnormal activation of the coagulation system occurs, involving platelet adhesion and aggregation via von Willebrand factor and clotting factors, leading to microthrombosis and ultimately DIC, which exacerbates systemic pathological progression (Jiang et al., 2020). Thus, the background of immunosenescence (represented by advanced age) and the active thromboinflammatory state (represented by high MPV) constitute synergistic pathways leading to adverse patient outcomes.

Beyond D-D, which was included in the earlier model, the XGBoost-based prognostic model identified new dimensional indicators with stronger predictive power. Elevated CK often indicates rhabdomyolysis or widespread tissue/cellular damage due to sepsis, reflecting infection severity and its direct cytotoxic effects, an end-organ damage indicator not fully captured in the model. The significance of PCT, a classic inflammatory marker for systemic bacterial infection, confirms that uncontrolled inflammatory storms are a core driving force ultimately leading to patient death.

The prognostic prediction model constructed in this study is both interconnected with and distinct from the previously developed infection risk model. Both incorporate indicators reflecting baseline vulnerability, coagulation dysfunction, and organ damage/metabolism. However, the differences in their variable combinations precisely reflect the nature of different clinical questions: the model focuses on identifying who is more likely to get infected, thus incorporating indicators representing pre-infection baseline states such as nutritional status and renal functional reserve. The prognostic model, in contrast, focuses on predicting the outcome for those already infected, thus emphasizing dynamic process indicators post-infection, such as the intensity of the inflammatory burst, acute coagulation disorders, and acute tissue injury.

The XGBoost model demonstrated superior predictive performance by capturing complex non-linear relationships and interactions among variables, a key advantage over traditional logistic regression. While logistic regression provides interpretable insights into static risk factors, XGBoost identifies dynamic, multi-dimensional feature combinations (e.g., ALB, D-D, UA) that more closely reflect the evolving pathophysiology of CRKP infection. Together, these complementary approaches balance interpretability with predictive accuracy, offering a more comprehensive foundation for clinical decision-making.

Several limitations of this study should be acknowledged. First, as a single-center retrospective study conducted at a single institution, the generalizability of our findings to other populations or healthcare settings may be limited. Second, the relatively modest sample size may have reduced statistical power for detecting additional risk factors and precluded meaningful subgroup analyses based on infection site or comorbidity type. Third, the retrospective design introduces potential selection bias, as patients with missing key data were excluded, and unmeasured confounding variables could not be accounted for. Fourth, although we employed internal validation techniques including 5-fold cross-validation and bootstrapping, the absence of external validation in independent cohorts means that our models’ performance metrics may be optimistic and may not reflect real-world performance in diverse clinical settings. Fifth, age emerged as a dominant predictor across both infection risk and prognostic models; while this reflects the strong biological impact of immunosenescence, it may also mask the contribution of other clinically relevant factors. Sixth, we did not perform direct comparisons with other machine learning algorithms such as Support Vector Machines or Random Forest, leaving open the possibility that alternative approaches might achieve comparable or superior performance. Future studies should validate our models in multicenter cohorts across diverse geographic regions to assess generalizability. Prospective designs with standardized data collection would minimize bias and capture time-varying factors (e.g., antibiotic timing). Larger samples would enable subgroup analyses by infection site and comorbidity. Comparative evaluations of multiple machine learning algorithms could identify optimal modeling strategies. Finally, implementation studies are needed to evaluate real-world impact on clinical decision-making and patient outcomes.

In summary, this study not only validated independent risk factors for CRKP infection using traditional statistical models but also leveraged advanced machine learning algorithms to develop tools with superior predictive performance. These predictors are deeply rooted in the host’s immune, nutritional, coagulation, and organ functional status, providing a crucial theoretical foundation and practical tools for the early clinical identification of high-risk patients, timely intervention, and optimization of antimicrobial strategies. Future research should focus on validating these models in prospective cohorts and exploring their integration into clinical decision support systems to ultimately improve patient outcomes.

## 5 Conclusion

This study employed the XGBoost algorithm to identify five key variables—age, albumin, D-dimer, creatinine, and uric acid—as the most important predictors of CRKP infection risk. The XGBoost model demonstrated superior predictive performance for both infection risk and mortality compared to traditional logistic regression. For clinical practice, routine assessment of these five factors is recommended to facilitate the early identification of patients at high risk of CRKP infection. These findings provide a data-driven basis for optimizing antimicrobial stewardship and implementing targeted interventions in high-risk populations.

## Data Availability

The raw data supporting the conclusions of this article will be made available by the authors, without undue reservation.
