# Supplementary material for: Machine learning-based prediction of carbapenem-resistant Klebsiella pneumoniae infection risk and prognosis
Source: Front Pharmacol. 2026 Mar 23;17:1780002. doi: 10.3389/fphar.2026.1780002 (PMC13050907; doi:10.3389/fphar.2026.1780002)
Supplement: Supplementary file 1 [file Table1.docx]

Table S1. Data Collection Parameters

| Category | Parameters Collected |
| --- | --- |
| Demographic Characteristics | Age, gender |
| Clinical Outcomes | 30-day, 60-day, 90-day, and 180-day mortality status |
| Microbiological Data | Pathogen identification (CRKP/CSKP), specimen type, antimicrobial susceptibility testing results |
| Blood Routine | RBC, WBC, PLT, MON%, LYM%, EOS%, BAS%, NEU%, HGB, HCT, MCH, MCHC, MCV, MPV, PDW, RDW |
| Liver Function | ALT, AST, ALB, TP, TBIL |
| Renal Function | UA, UREA, CREA |
| Cardiac Biomarkers | LDH, HBDH, CK, CK-MB |
| Electrolytes and Metabolism | GLU, K, Na, CO2CP |
| Inflammatory Markers | PCT, IL-6, CRP |
| Coagulation Panel | PT, PT-INR, PTA, TT, APTT, AT3, D-D, FBG, FDP |

Table S2. Package Selection

| Package | Primary Function | Selection Rationale |
| --- | --- | --- |
| xgboost | Machine learning (XGBoost) | Efficient gradient boosting implementation with built-in cross-validation and regularization |
| glmnet | Logistic regression | Lasso regularization for variable selection and multicollinearity handling |
| pROC | ROC analysis | Comprehensive ROC visualization with bootstrapped confidence intervals |
| caret | Data partitioning | Stratified data splitting for consistent outcome distribution |
| SHAPforxgboost | Model interpretation | SHAP value computation for global and individual-level explanation |
| rmda | Decision curve analysis | Clinical utility assessment across threshold probabilities |
| rms | Calibration assessment | Bias-corrected calibration curves with bootstrapping |
| tableone | Descriptive statistics | Efficient generation of well-formatted baseline characteristic tables |
